# Supplementary material for: Genomic Differences and Mutations in Epidemic Orf Virus and Vaccine Strains: Implications for Improving Orf Virus Vaccines
Source: Vet Sci. 2024 Dec 2;11(12):617. doi: 10.3390/vetsci11120617 (PMC11680149; doi:10.3390/vetsci11120617)
Supplement: Supplementary file 1 [file vetsci-11-00617-s001.zip › vetsci-3276087-supplementary.pdf]

Original Images:

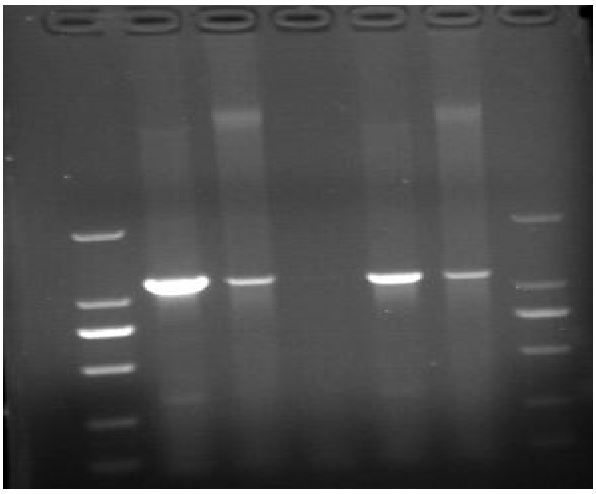

Figure Legends:

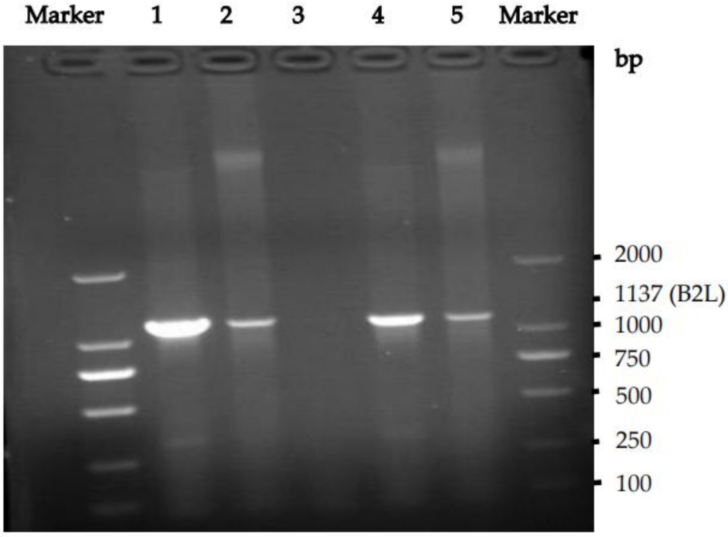

Figure 1. Positive amplification products of B2L (1137 bp) were obtained via polymerase chain reaction. Lanes 1–3: ORFV-1V, ORFV-2W, and a negative control, respectively. Lanes 4–5: ORFV-1V and ORFV-2W, respectively. The marker shown is a 2000-bp DNA marker.
