# Supplementary material for: Genomic Differences and Mutations in Epidemic Orf Virus and Vaccine Strains: Implications for Improving Orf Virus Vaccines
Source: Vet Sci. 2024 Dec 2;11(12):617. doi: 10.3390/vetsci11120617 (PMC11680149; doi:10.3390/vetsci11120617)
Supplement: Supplementary file 1 [file vetsci-11-00617-s001.zip › vetsci-3276087 table S1.pdf]

Table S1. Single nucleotide polymorphisms analysis of ORFV-1V

Strain	Variant type	Gene	Strand	NT	AA	Effect	Product
ORFV-1V	Snp (missense)	*ORF067*	+	586/699	196/232	586 G>C, Asp 196 His	Uracil DNA glycosidase
	Snp (missense)	*ORF072*	–	337/1917	113/638	337 A>G, Ile 113 Val	Transcription termination factor NPH-I
	Ins (frameshift)	*ORF102*	–	442/1575	148/524	442 dup A, Asn 148 fs	A-type inclusion protein/fusion peptide hybrid

Abbreviations: "NT" Nucleotide. "AA" Amino acid. "+" Plus. "–" Minus.
